# Gesture Influences Resolution of Ambiguous Statements of Neutral and Moral Preferences

**DOI:** 10.3389/fpsyg.2020.587129

**Published:** 2020-12-10

**Authors:** Jennifer Hinnell, Fey Parrill

**Affiliations:** ^1^Department of English Language and Literatures, The University of British Columbia, Vancouver, BC, Canada; ^2^Department of Cognitive Science, Case Western Reserve University, Cleveland, OH, United States

**Keywords:** cohesive gesture, co-speech gesture, reference resolution, preference, contrast, discourse, multimodal communication, moral issues

## Abstract

When faced with an ambiguous pronoun, comprehenders use both multimodal cues (e.g., gestures) and linguistic cues to identify the antecedent. While research has shown that gestures facilitate language comprehension, improve reference tracking, and influence the interpretation of ambiguous pronouns, literature on reference resolution suggests that a wide set of linguistic constraints influences the successful resolution of ambiguous pronouns and that linguistic cues are more powerful than some multimodal cues. To address the outstanding question of the importance of gesture as a cue in reference resolution relative to cues in the speech signal, we have previously investigated the comprehension of contrastive gestures that indexed abstract referents – in this case expressions of personal preference – and found that such gestures did facilitate the resolution of ambiguous statements of preference. In this study, we extend this work to investigate whether the effect of gesture on resolution is diminished when the gesture indexes a statement that is less likely to be interpreted as the correct referent. Participants watched videos in which a speaker contrasted two ideas that were either neutral (e.g., whether to take the train to a ballgame or drive) or moral (e.g., human cloning is (un)acceptable). A gesture to the left or right side co-occurred with speech expressing each position. In gesture-disambiguating trials, an ambiguous phrase (e.g., *I agree with that*, where *that* is ambiguous) was accompanied by a gesture to one side or the other. In gesture non-disambiguating trials, no third gesture occurred with the ambiguous phrase. Participants were more likely to choose the idea accompanied by gesture as the stimulus speaker’s preference. We found no effect of scenario type. Regardless of whether the linguistic cue expressed a view that was morally charged or neutral, observers used gesture to understand the speaker’s opinion. This finding contributes to our understanding of the strength and range of cues, both linguistic and multimodal, that listeners use to resolve ambiguous references.

## Introduction

One only has to look around a room full of people spending time together to see that language consists of more than words on a page or a highly patterned audio signal. In face-to-face interaction, speakers are rarely still. Rather, in addition to the speech sounds normally associated with language, they also move their hands, shoulders, head, and manipulate their facial expressions in ways that are semantically and temporally aligned with their speech. Studies of language and cognition have thus moved beyond text and speech to include these movements as critical contributors to linguistic meaning-making (Kita, 2000, 2003; McClave, 2000; Müller et al., 2013, 2014; Levinson and Holler, 2014; Enfield, 2017; Feyaerts et al., 2017; Kita et al., 2017).

The manual gestures that speakers use in addition to speech to communicate their message are known as co-speech gestures. These gestures can be idiosyncratic and *ad hoc*, functioning “now in one way, now in another” (Kendon, 2004: 225) depending on the context. However, they are also characterized by a high degree of regularity in features such as the gesture form (Kendon, 2004; Müller, 2004), duration (Duncan, 2002), and timing of gesture related to speech (Kelly et al., 2010, 2015; Church et al., 2014; Hinnell, 2018). For example, the palm-up open-hand (PUOH) gesture is one example of a form that exhibits a stable form-meaning pairing across a speech community (Ladewig, 2014; Müller, 2017). The handshape and orientation of the PUOH are stable and iconically represent its meaning of presenting or giving information (with the open palm held in such a way as to potentially hold a small object). Similarly, a holding away gesture is prototypically enacted with both palms facing forward and raised vertically in front of the speaker; the form iconically represents how it is used, namely “to establish a barrier, push back, or hold back” a line of action, e.g., to reject topics of talk (Bressem and Müller, 2014, p. 1593; see also Kendon, 2004).

Importantly for the research presented here, speakers also use the space around their bodies in which they gesture – known as gesture space (McNeill, 1992; Priesters and Mittelberg, 2013) – in highly systematic ways to anchor objects, ideas, and other discourse elements. For example, when a speaker describes a past event and mentions that an object in the room was to the right of her, she will most likely indicate the object using a gesture to the right of her body. That is, speakers gesture in the space around their bodies to locate the things they are talking about, and, importantly, the locations of these objects in the gesture space reflect the locations of the objects in the real world. For example, we know that speakers gesture about concrete referents (objects, characters, locations) in locations in gesture space that are consistent with real locations they recall from pictures, videos, remembered events, etc. (So et al., 2009; Perniss and Ozyürek, 2015). In addition to assisting the speaker in tracking referents and building coherent discourse (McNeill, 2005; Gullberg, 2006), it’s been suggested that this allows observers to use the spatial information contained in gesture to track referents and also increases comprehension (Gunter et al., 2015; Sekine and Kita, 2017).

**FIGURE 1:**
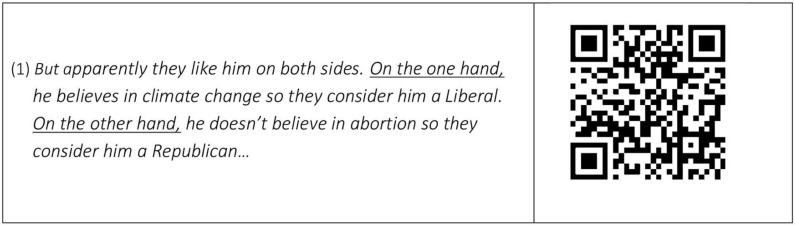
Contrastive use of gesture. 2015-09-24_1700_US_KABC_The_View, 191–201. Red Hen dataset http://redhenlab.org (click here or scan QR code to view the video clip; Uhrig, 2020).

The systematic use of gesture space also extends to abstract referents, e.g., ideas, emotions, and discourse elements (Parrill and Stec, 2017). A corpus study of English contrastive gestures showed that speakers regularly produce gestures to each side of space when contrasting two ideas (Hinnell, 2019). For example, when speakers use fixed expressions that contrast two abstract concepts, such as *on the one hand/on the other hand* or *better than/worse than*, they regularly produced gestures to each side of their body that reflect this contrastive setup, as shown in Figure 1. Finally, the role of space in expressing contrast extends to signed languages. For example, in American Sign Language, signers build a spatial map to make comparisons (Winston, 1996; Janzen, 2012). This comparative spatial mapping strategy has both a referential function and is used to structure discourse (Winston, 1996, p. 10).

In addition to these studies of how speakers produce gesture in contrastive discourse, experimental work has investigated how the use of gesture and gesture space affects a participant’s language comprehension. Gestures that are used in establishing locations for and then tracking references in discourse are known as cohesive gestures (McNeill, 1992). It’s been shown that when cohesive gestures co-occur with congruent speech, they facilitate language comprehension (Gunter et al., 2015) and can influence the interpretation of ambiguous pronouns (Goodrich Smith and Hudson Kam, 2012; Nappa and Arnold, 2014). The effect of gestures that locate referents in spatial locations extends even in the absence of the gesture. Sekine and Kita (2017) showed that listeners build a spatial representation of a story and that this representation remains active in subsequent discourse. In their study, participants were presented with a three-sentence discourse involving two protagonists. Video clips showed gestures locating the two protagonists on either side of the gesture space in the first two sentences. The third sentence referred to one of the protagonists, which could be inferred by a gendered pronoun but, importantly, did not co-occur with gesture. The name of the protagonists appeared on the screen and participants were asked to respond with one of two keys to indicate which protagonist was referred to. In the condition in which the name appeared on the side that was congruent with the gestures, participants performed better on the stimulus-response compatibility task. Importantly, there was no strategic advantage to the listeners to process the cohesive gestures, as the speech provided all information that was useful to the task (i.e., gender of protagonists). This finding extends previous findings (e.g., Goodrich Smith and Hudson Kam, 2012) that cohesive gestures allow listeners to build spatial story representations and demonstrates that listeners can “maintain the representations in a subsequent sentence without further gestural cues” (ibid: 94). In sum, listeners use a speaker’s cohesive gestures to build spatial representations of concrete entities such as people or objects. This process occurs quickly (i.e., with each location mentioned or gestured once to establish a referent in a location and once again to refer back to it) and the representation remains active over the course of subsequent discourse.

Less is known about the effect on comprehension and reference resolution of gestures that contrast abstract ideas, rather than entities in narrative tasks as in the comprehension experiments described above. In previous work, Parrill and Hinnell (in review) found that observers use gesture to resolve an ambiguous statement of preference between two contrasting ideas in the same way they use gesture to resolve ambiguous references such as pronouns referring to concrete entities. That is, we found that when a speaker accompanies a statement of preference with a gesture to the same side of the gesture space that the idea was originally anchored in, the listener more frequently interprets the speaker’s preference to be that idea. This suggests that people use gesture to build a spatial representation and that this representation aids listeners in resolving ambiguous references in contrastive scenarios and contributes to their understanding of a speaker’s preference.

The robust literature on reference resolution provides evidence that a wide set of linguistic constraints influences the successful resolution of ambiguous pronouns. Known constraints on a listener’s pronoun interpretation include linguistic salience, or conceptual accessibility. An example of linguistic salience is the subject or first-mention bias, which captures the fact that speakers most often assume the first-mentioned reference to be the referent of the ambiguous pronoun (e.g., *Francis* in the ambiguous sentence pair, *Francis went shopping with Leanne. She bought shoes*) (Gernsbacher and Hargreaves, 1988; Nappa and Arnold, 2014). Focus constructions (Arnold, 1998; Cowles et al., 2007) and recent mention (Arnold, 2001, 2010) are other linguistic constraints on reference resolution (see review in Arnold et al., 2018). Models such as Bayesian models are also based on the notion of salience. Such models suggest that reference resolution is based on probability estimates that a listener calculates based on semantic knowledge (Hartshorne et al., 2015) or from their experience of how linguistics units are used, e.g., that speakers “tend to continue talking about recently mentioned entities, especially subjects” (Arnold et al., 2018: 42; see also Arnold, 2001, 2010). As the gesture literature cited above reveals, non-verbal cues also influence pronoun interpretation; however, studies have shown that linguistic cues trump non-verbal cues during pronoun interpretation, e.g., Arnold et al. (2018) provide evidence that people rely more on their prior linguistic experience (as assessed by reading experience) than on eye-gaze aligned with the referent of the pronoun.

In light of this evidence regarding both referent tracking in multimodal contexts and reference resolution more generally, in the current study we investigate the role of gesture during the interpretation of referentially ambiguous expressions to address the relative importance of gesture as a cue in reference resolution relative to cues in the speech signal. We go beyond current literature, which has examined how gesture and gesture space are used to track concrete information (such as two entities in narrative space), to investigate the tracking of contrastive abstract information (such as pairs of moral statements). We assess whether the effect of gesture on resolving ambiguous statements is diminished when the gesture indexes a statement in speech that is less likely to be interpreted as the correct referent (e.g., a morally reprehensible position).

In this study, participants were presented with video scenarios in which the stimulus speaker contrasted two ideas. The stimulus speaker made a gesture to the left or right side that co-occurred with speech expressing each idea. Scenarios were either neutral (e.g., whether to take the train to a ball game or drive) or moral (i.e., likely to evoke strong feelings, as in human cloning is acceptable). We created two trials in which gestures were varied in the following way: in gesture-disambiguating trials, an ambiguous phrase (e.g., *I agree with that*, where *that* could refer to either previously expressed idea^1^) was accompanied by a gesture to one side or the other; in gesture non-disambiguating trials, no third gesture occurred with the ambiguous phrase. Participants were asked to identify the stimulus speaker’s preference and were also asked to record their own personal preference. We explore whether participants are more likely to choose the idea accompanied by gesture as the stimulus speaker’s preference (as found in earlier work), and whether this pattern changes as a function of scenario type (i.e., whether the items being contrasted were neutral in nature or involved questions of morality). We compare moral vs. neutral statements to assess whether one’s own belief or that of the speaker can compete with, and potentially override, a contrastive statement of preference that is reinforced by gesture. Participants are more likely to have strong views about moral statements than about neutral statements.

This approach of considering the effect of a participant’s own views on their resolution of ambiguous preference statements also aligns with an interactional approach that is gaining prominence in cognitive linguistics that considers meaning as a coordinated process between interlocutors (Clark, 1996; Du Bois, 2007; Mondada, 2013; Brône et al., 2017; Feyaerts et al., 2017). We therefore explore to what extent the participant’s preference impacts the role of a co-occurring gesture on a preference statement in a contrastive scenario.

In line with literature on the role of gesture in expressing contrast and resolving ambiguous references, we hypothesized that in situations where a gesture co-occurs with one element of the contrast and then re-occurs in that place with the expression of the speaker’s preference, participants would be more likely to assess this element as the speaker’s preference in the scenario. Furthermore, in assessing the impact of a participants’ moral views on this effect, we hypothesized that in cases where the participant disagreed strongly with the morally unacceptable position (e.g., slavery construed positively), this effect of the speaker’s gesture would decrease. That is, the participant’s own views would interact with the confirming effect of the gesture on how the participant assessed the speaker’s preference.

The findings contribute to an understanding of the degree to which factors beyond linguistic constraints play a role in reference resolution. As cited above, Arnold et al. (2018) found gaze played less of a role than linguistic constraints in reference resolution. Here, we explore whether gesture is a powerful enough cue to resist countervailing information such as a morally abhorrent position. As such, the study contributes to our understanding of the range of cues, both linguistic and multimodal, that people recruit to resolve ambiguous references.

## Materials and Methods

### Design and Predictions

**FIGURE 2:**
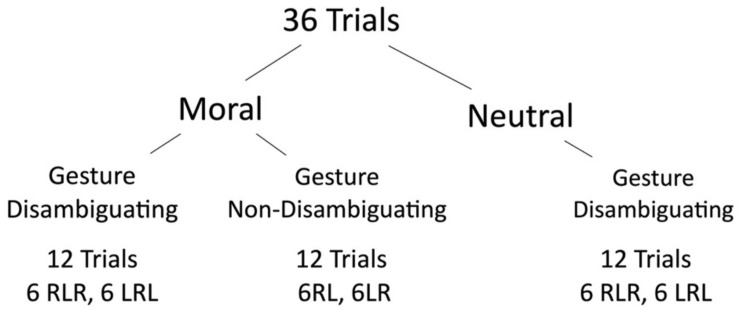
Experimental design.

We carried out a within-participants study examining the impact of two factors, scenario type (neutral, moral) and gesture trial type (gesture disambiguating, or GD; gesture non-disambiguating, or GND), on the frequency of choosing the first element of the contrast (e.g., statement A, if the contrast was A *but* B) for stimulus speaker preference (see Figure 2 below). For the moral scenario type, the A statement always expressed the morally unacceptable option. In our earlier study (Parrill and Hinnell, in review), speakers showed a clear bias to choosing the last mentioned referent as the speaker’s preference in a pair of concessive statements. Thus, the A statement was less likely to be predicted as speaker’s preference. Furthermore, we operated on the assumption that having the A statement express the morally unacceptable position rendered the referent more predictable, as the B statement was more likely to represent the speaker’s intended position. We also predicted that participants would be more likely to choose the A statement for stimulus speaker preference when the speaker makes a disambiguating gesture, i.e., for GD trials as compared to GND trials. If it is the case that gesture plays less of a role when the majority of participants disagree with the position expressed in the A statement, then we would expect the frequency of those choosing A to decrease for moral scenarios as compared to neutral scenarios within the GD trials.

### Materials

We created 36 scenarios, each containing the following elements:

(1) An attitude about a topic (A statement).
(2) The concessive “but.”
(3) A differing attitude about the topic (B statement).
(4) A hedge indicating uncertainty.
(5) An ambiguous statement indicating a preference for either the A or B statement^2^.

For example, “My little brother’s not on Facebook because he thinks it’s a waste of time” (A statement), “but” (concessive) “my other brother says he can’t do job networking without it” (B statement). “I can see what they’re getting at, but” (hedge) “I think he’s right” (preference statement). The preference statement is ambiguous because “he” could refer to either “little brother” or “other brother.”

We created two types of scenarios, neutral and moral. For neutral scenarios, we used previous research (Parrill and Hinnell, in review) as a starting point. We selected twelve scenarios for which participants in the previous study chose the A and B statements at about equal rates when asked about their own personal preference. Returning to the example given above, about half the participants in our previous study thought Facebook is a waste of time and about half thought Facebook is useful. We created 24 moral scenarios based on topics selected from Gallup’s annual Values and Beliefs poll (Jones, 2017) and a study of divisive social issues (Simons and Green, 2018). Topics were included if at least 70% of participants in these sources considered one position related to the topic morally unacceptable. We then created scenarios about these topics. Moral scenarios always had the following form: An A statement that expressed the morally *unacceptable* position, the concessive “but,” a B statement that expressed the morally *acceptable* position, a hedge, and an ambiguous preference statement. For example, 86% of participants in the Gallup study considered human cloning morally unacceptable, so human cloning was included as a topic. An example scenario is: “Shelley was saying if we can clone humans, we can fix genetic disorders and end suffering” (A statement, morally unacceptable position), “but” (concessive) “Alicia was saying there’s never a good reason to go down that path” (B statement, morally acceptable position). “It’s tough to say, but” (hedge) “I guess I agree with her” (preference statement). The preference statement is ambiguous because “her” could refer to either Shelly or Alicia.

We first recorded audio for each scenario. The first author read each scenario as naturally as possible. For the recording of video, a research assistant was instructed to sit in a comfortable posture and to perform (speak and gesture) several scenarios as naturally as possible. Scenarios were performed in two different ways: a gesture-disambiguating version (GD) and a gesture non-disambiguating version (GND).

**FIGURE 3:**
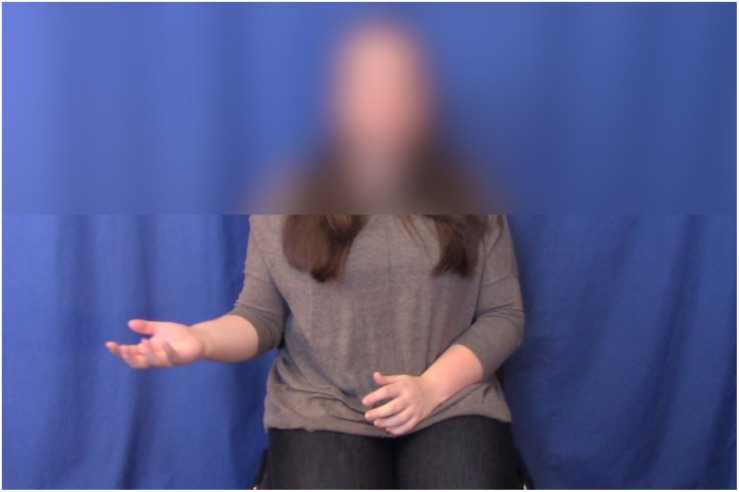
Example stimulus.

Both the GD and GND versions of the video featured palm-up open-hand gestures (see Figure 3). These were performed with the A and B statements^3^. The research assistant performed versions with the left hand first and with the right hand first. For the GND version, the speaker sat still and did not gesture during the preference statement. For the GD version, the speaker performed a final palm-up open-hand gesture with the preference statement. The final gesture always occurred in the location where the A statement gesture had been performed. For example, if the first gesture was on the left, the final gesture would be performed on the left as well.

We created four types of videos: (1) right hand first, left hand second, final gesture with right hand, (2) left hand first, right hand second, final gesture with left hand, (3) right hand first, left hand second, no third gesture, and (4) left hand first, right hand second, no third gesture. Using Final Cut Pro, we matched audio clips to these four different videos to create two stimulus lists. We used stimulus lists to minimize the chances that specific properties of the scenarios would impact our results.

Scenarios were assigned to GD and GND videos to create 12 moral GND trials and 12 moral GD trials. Because our previous study indicated that we could not include more than 36 trials without participants becoming fatigued, we created only GD neutral trials. This design was selected to maximize our ability to compare *moral* scenarios across gesture disambiguating and non-disambiguating trials, without the study lasting so long that participants would not be able to attend to the stimuli. We elected to use a smaller number of neutral scenarios, and to use only GD trials for our neutral scenarios, because our previous work indicated that without gesture, participants will choose the B statement as the speaker’s preference at a rate above chance (about 70%). Moral scenarios were counterbalanced across stimulus lists so that each occurred with both GD and GND videos.

When adding audio to video, we aligned specific auditory and gestural features. Gesture strokes (the effortful, meaningful portion of a gesture: McNeill, 1992) were aligned with the subject noun phrases (e.g., “little brother,” “other brother”). The stroke of the final gesture for GD stimuli was aligned with the ambiguous noun phrase (e.g., “he’s”). We used Final Cut Pro to blur the speaker’s face and upper shoulders so that mouth movements did not reveal the fact that audio and video had been edited, as shown in Figure 3. We also did this masking so that facial expressions and head movements would not affect participants’ judgments. There was some variation in intonational contours and in the research assistant’s posture across different videos. This was desirable, as it made the scenarios feel more natural.

In summary, the outcome of the editing was to create two versions of each scenario, with scenarios randomly paired to GD and GND videos for the moral scenarios, and always paired with GD videos for the neutral scenarios. Within moral and neutral categories, scenarios were randomly paired with videos in which the right versus left hand was used first. Audio and video were carefully aligned to preserve the systematicity of auditory and gestural cues. Participants were presented with both neutral and moral scenarios and both GD and GDN (a within-participants study). Trials were presented in random order.

### Procedure

After an informed consent/instruction screen, participants were presented with a scenario. After viewing each scenario, participants responded to a question asking for their judgment about the stimulus speaker’s preference. The exact question was matched to the preference statement, so that, for example, a preference statement ending with “I think he’s right” would be followed by a question asking “who does the speaker think is right?” Participants chose between options matched to the scenario, such as “Facebook is a waste of time” and “Facebook is needed for networking.” Options were presented horizontally, and their locations were random (thus, the option appearing to the left was random for each trial so that the choice options didn’t necessarily match the spatial location of the A and B statements). Second, participants responded to the question “What is your personal opinion/preference?” and were presented with the same options as in the previous variable (e.g., “Facebook is a waste of time,” “Facebook is needed for networking”). As with the previous response, the location of options was randomized with respect to location. Responses to these two questions serve as our dependent variables and will be referred to as “stimulus speaker preference” and “participant preference.” After the last scenario, participants answered demographic questions about gender (male, female, other), race, age, fluency in a second language, political ideology (“do you identify as more progressive/more conservative”), and participants were asked “what do you think this study was about?” (open entry).

### Participants

Eighty participants were recruited using the online data collection platform Amazon Mechanical Turk. Mechanical Turk data have been shown to be comparable to data collected in academic research studies, but the Mechanical Turk population is more diverse in age, education, and race/ethnicity than most typical university research populations (Burhmester et al., 2011). Participants were required to be within the United States to take part and were compensated with $3.50. The study took about half an hour for participants to complete.

## Results

Data have been uploaded to Open Science Framework and can be found here: https://osf.io/t3sbx/. Data were examined to ensure no participants completed the study too quickly to have done the task correctly. One participant was removed from the List 1 data for this reason. Demographic details (age, gender, race, political affiliation) are presented in the Supplementary Appendix 1. When asked about the topic of the study, only six of the 80 participants said that the study was about gesture or body language (these six were not removed from the analyses). The majority of participants said that the study was about things like persuasion, decision making, or opinions. Data were analyzed using R version 4.0.0 (R Core Team, 2020). Utility packages used for data manipulation, cleaning, and analysis include dplyr (Wickham et al., 2020), tidyr (Wickham and Henry, 2020), psych (Revelle, 2019), car (Fox and Weisberg, 2019), lawstat (Hui et al., 2008), and DescTools (Signorell et al., 2020).

We present the proportion of participants who chose the A statement as their own personal preference/opinion for each scenario in the Supplementary Appendix 2 along with the scenario texts. In general, the scenarios patterned as expected (neutral scenarios around 50%, moral scenarios below 30%). There were some exceptions (to which we will return in the discussion), but this is not problematic for our predictions. The majority of the scenarios behaved as expected and we examine frequency data.

Because our data are categorical and do not meet the assumptions required for parametric tests (they are non-normal, non-interval, and we do not have homogeneity of variance), we used chi-square analyses to answer our research questions. These analyses mean that we will not examine some possible relationships (how different scenarios might pattern, variability contributed by participants, etc.). However, these analyses were preferable to logistic regression as they require fewer assumptions about the data and are simpler to interpret.

**FIGURE 4:**
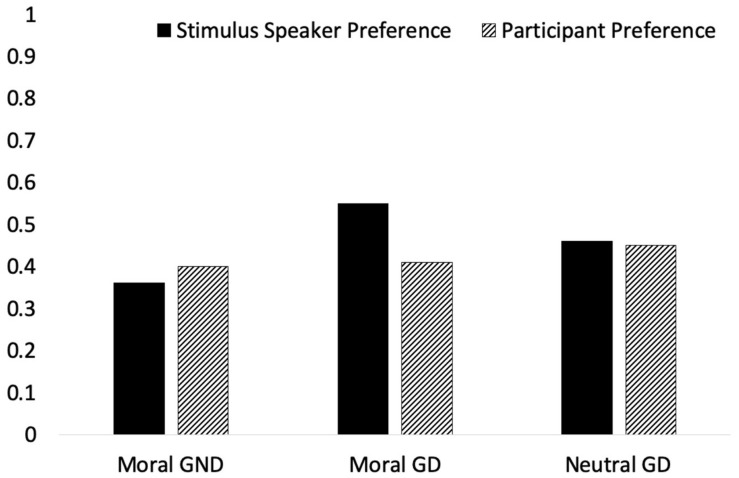
Proportion of participants who chose the A statement by condition and scenario (no error bars shown because the figure shows overall proportions).

Figure 4 shows the proportion of participants who chose the A statement for stimulus speaker preference. Table 1 shows an overall picture of the data both as frequencies and proportions according to scenario type and trial type. The key comparison is between the proportion of participants choosing the A statement for stimulus speaker preference. This proportion is higher for both types of GD trials (46% and 55%) compared to GND trials (36%).

**TABLE 1:** Responses by scenario type and trial type (proportions in parentheses).

Scenario type	Trial type	Response*	Stimulus speaker preference	Participant preference
Neutral	GD	A	436 (0.46)	423 (0.45)
		B	512 (0.54)	525 (0.55)
Moral	GND	A	345 (0.36)	376 (0.40)
		B	603 (0.60)	572 (0.60)
	GD	A	524 (0.55)	385 (0.41)
		B	424 (0.45)	563 (0.59)

*For moral scenario types, A was the morally unacceptable response, B was the morally acceptable response.

Table 2 presents responses according to what the participant selected for both dependent variables, by trial type and list. That is, 117 participants chose A for both stimulus speaker preference and participant preference for the Moral GND trials for list 1. While this presentation of the data is not as easy to relate to the research questions as Table 1, the contingency tables created allow us to use a variant of the chi-square test that accounts for multiple dimensions, called the Cochran-Mantel-Haenszel chi-square test. This test creates a common odds ratio (OR) across multiple contingency tables, which allows researchers to avoid Simpson’s paradox (Simpson, 1951), wherein patterns that appear when comparing one subset of the data disappear when comparing another subset. ORs are a conditional estimate of the extent to which a treatment impacts an outcome (e.g., the odds of choosing the A statement for stimulus speaker preference given you chose A for participant preference). An OR close to 1 indicates no impact on outcome (outcome is 1 time as likely). Overall, these analyses test a null hypothesis that the choice between A and B for stimulus speaker preference is not independent of the choice for participant preference.

**TABLE 2:** Stimulus speaker and participant preference by trial type and list, with odds ratios.

	Stimulus speaker preference*	Participant preference		Odds ratio**
		
		A	B	
Moral, GND, List 1	A	117	72	4.52
	B	77	214	
Moral, GND, List 2	A	111	58	6.17
	B	80	258	
Moral, GD, List 1	A	145	126	3.66
	B	50	159	
Moral, GD, List 2	A	125	128	2.52
	B	60	155	
Neutral, GD, List 1	A	141	118	2.63
	B	69	152	
Neutral, GD, List 2	A	136	117	2.08
	B	77	138	

*For moral scenario types, A was the morally unacceptable response, B was the morally acceptable response.

**Odds ratio presents the odds of choosing B for participant preference given person chose B for stimulus speaker preference.

The CMH statistic of 228.45 (1), *p* < 0.0001 (pooled OR = 3.26) indicates a significant association between one of our variables and outcomes. This leads us to reject the null hypothesis that the dimensions are independent. We then tested the homogeneity of ORs using the Breslow-Day test, which tests the null hypothesis that the ORs are all statistically the same. R’s DescTools allows the Breslow-Day test to be calculated with or without Tarone’s adjustment; we opted to calculate without because we have a relatively large sample size and the need for more accurate *p*-values was moot. The Breslow-Day chi-square statistic [X2 (5, *N* = 2883) = 21.14, *p* = 0.0008] indicates that the ORs are not the same.

Table 2 shows the individual ORs for each by-list contingency table. In general, participants tend to choose B for both dependent variables (that is, they “pile up” in the B/B corner of the tables). The odds of this are particularly high when there is no disambiguating gesture (between 4 and 6 times as likely).

To provide some statistical information about the impact of list, we compared the two moral GD contingency tables (that is, across list 1 and list 2). Here the Breslow-Day chi-square statistic [X2 (1) = 1.73, *p* = 0.19] requires us to fail to reject the null hypothesis that the two ORs are statistically equivalent. This indicates that the association is not based on list for moral GD trials. A comparison of the two moral GND contingency tables across list also requires us to fail to reject the null hypothesis that the two ORs are the same [X2 (1) = 1.18, *p* = 0.28]. This indicates that the association is not based on list for moral GND trials. Finally, a comparison of the two neutral GD contingency tables (across lists) also requires us to fail to reject the null hypothesis that the two ORs are the same [X2 (1) = 0.75, *p* = 0.39]. This indicates that the association is not based on list for neutral GD trials. Taken together, this set of analyses indicates that the lists can be collapsed, thus we aggregated the data across lists.

To determine the impact of trial type (with a final disambiguating gesture, without a final gesture), we first compared moral GND to moral GD trials. The Breslow-Day chi-square statistic indicates that there is an association between trial type and outcome [X2 (1) = 7.56, *p* = 0.006]. For moral scenarios, participants were more likely to choose the A statement when a gesture was produced on the “A side” with the preference statement, compared to when there was no gesture.

To understand the impact of scenario type (moral, neutral), we compared moral GD trials to neutral GD trials. The Breslow-Day chi-square statistic indicates that there was no association between scenario type and outcome [X2 (1) = 1.77, *p* = 0.18]. Participants were equally likely to choose the A statement for moral GD and neutral GD trials.

Finally, we verified that gesture was used to disambiguate preference across scenario types by comparing the moral GND trials to the neutral GD trials. The Breslow-Day chi-square statistic indicates that there is an association between scenario type and outcome [X2 (1) = 17.08, *p* < 0.00001]. Participants were more likely to choose the A statement when a gesture was produced on the “A side” with the preference statement (neutral GD trials), compared to when there was no gesture (moral GND trials).

## Discussion

We predicted that when presented with scenarios in which the speaker produced an ambiguous expression of preference, participants would use gesture to disambiguate, if gesture was available. That is, if the speaker produced a gesture in the location where she had previously gestured when presenting a position, participants would be more likely to assume she preferred that option. This prediction was supported. Participants were more likely to choose the A statement when a gesture was produced in the “A location” during the preference statement. This replicates our previous work, showing that gesture is integrated into participants’ understanding of a speaker’s preference. In the context of research on cohesive gestures and reference resolution, this finding provides further evidence that gesture is recruited by the listener to resolve ambiguous references.

Beyond this, we extended our previous work by asking whether gesture as a cue in reference resolution would play less of a role when the position expressed in the A statement was an unpopular one. That is, if the speaker appeared to indicate via the location of her gesture that she was in favor of slavery, would participants be more likely to ignore her gesture and assume she preferred the more acceptable B statement position? In fact, we found no effect of scenario type. Participants were equally likely to choose the A statement when a gesture in the “A location” occurred with the preference statement regardless of whether the scenario was a moral or a neutral one. This finding suggests that gesture is a relatively strong referential cue, i.e., it can influence listeners to select an intended referent even when the referent indexes countervailing contextual information such as a morally unacceptable position.

While the presence of gesture shifted participants’ assessment of the speaker’s preference, participants still chose the B statement (whatever came last) between 40 and 60% of the time. In these cases, the linguistic cue appears to override the gestural cue. Even though a participant was using gesture to indicate a preference for a position that is relatively unpopular (i.e., in moral scenarios), the pattern was the same. Further studies are needed to explore whether gesture plays a more prominent role when the linguistic cue is weaker.

While Arnold et al. (2018) found that people rely more strongly on linguistic experience than on eye gaze, our findings suggest that contextual information such as a speaker’s predicted preference can indeed be “trumped” by gesture in the resolution of ambiguous reference. In our study, participants relied on the gestural cue for the morally unacceptable scenarios, despite most of the participants indicating they were not explicitly aware of the gestures, or at least of gesture as a point of the study. While not necessarily at odds with the finding of Arnold et al. (2018) given that here we examine the role of gesture rather than gaze, which may be a weaker cue, our findings underscore the need for further studies that include a range of linguistic, contextual, and multimodal cues to assess their relative strengths in reference resolution contexts.

Although the majority of our scenarios patterned the way we expected them to (that is, were neutral or moral, according to the way we operationalized these concepts for this project), there were some interesting exceptions. Participants in our data were more favorable toward human cloning, high unemployment, vandalism, air pollution, and polygamy than predicted. It is important to note that our scenarios justified a particular position (e.g., human cloning is good because it can end human suffering), whereas the research we were drawing from only presented a topic and asked participants to align as pro or con. It is also worth noting that 58% of our participants identified as politically conservative and that our data were collected in June, 2020. This was a highly atypical historical moment, as the United States was experiencing record unemployment due to the COVID-19 global pandemic in addition to sustained national protests over police brutality and racial injustice. This may have had some impact on responses to human cloning (as a means of curing disease), high unemployment, vandalism (framed as an act of protest in the scenario), and polygamy (framed as sharing the burden of childcare in the scenario). There were also two neutral scenarios where participants chose the A statement at rates considerably below 50%. Again, because our analyses are frequency based, these exceptions are not problematic, but do underscore the variability in opinion that makes such research challenging.

Another limitation of the current study was in the variability of the stimuli. Some of the preference statements included a second hedge within the preference statement, e.g., *I don’t know, but I guess I agree more with that*, as opposed to *hard to know, but his argument makes more sense to me.* Although this may have introduced more variability, this was done to incorporate the most natural speech possible in an experimental context; corpus studies have shown that speakers very frequently “stack” highly stanced elements such as hedges (Hinnell, 2019, 2020).

Another factor that impacted the naturalness of the stimuli was the decision to obscure the face of the speaker. This was done to remove the possibility of mouth movements revealing the fact that audio and video had been edited. Since gaze is frequently where interlocutors fixate when interacting with a speaker, this frequently used stimuli design may push the listener to pay more attention to the hands than they normally would [i.e., listeners tend not to attend to speakers gestures directly (Gullberg and Kita, 2009)]. We have attempted to mitigate the impact of this design somewhat through our debriefing process, in which we asked participants what they thought the study was about. Responses indicate that gesture was not very salient^4^. Finally, though we collected demographic data, we have not analyzed them in detail, planning instead to include them in future studies. It may be that additional patterns emerge when we examine sex, race, age, or political identification (though our measure of this was quite gross, being only a binary choice between more progressive and more conservative).

Several further questions remain. Firstly, in this study the stimulus speaker gestured only with PUOH gestures. However, the corpus studies in Hinnell (2019, 2020) suggest that speakers also regularly use other hand forms as well as other body articulators (e.g., head movements side to side) to indicate contrast, particularly when the referents are abstract. The question arises, then, whether other handshapes would affect the comprehension of contrastive gestures of preference and whether the effect is the same if the contrast is indicated in the head rather than the hands. That is, do hand form and articulator influence comprehension as well as placement in gesture space. Secondly, participants in this study were a variety of ages (mean age 36). Sekine and Kita (2015) have shown that children ∼5 years of age fail to integrate spoken discourse and cohesive use of space in gestures. We would expect that children of this age would also fail to integrate gestures of preference as explored in this study at that age, acquiring this ability before the age of 10 (in Sekine and Kita’s study, 10 year-olds performed the same as adults).

In sum, in this study we explored the effect of gesture on the observer in contrastive discourse, examining in particular the effect of gesture when speakers were expressing preference about neutral vs. highly moral issues. Findings suggest that gesture disambiguates an expression of the speaker’s preference for the observer. This contribution does not change even when the view being expressed is contrary to the participants’ beliefs and might be seen as socially unacceptable (e.g., the suggestion that slavery had benefits). These findings extend the scope of reference resolution studies beyond concrete referents in narrative storytelling to contrastive scenarios involving abstract referents. Furthermore, as one of few reference resolution studies to evaluate the strength of gesture in light of contextual cues, it points to the need to include multimodal cues in reference resolution studies and underscores the importance of gesture in creating multimodal discourse.

## Data Availability Statement

The datasets presented in this study can be found online at: https://osf.io/t3sbx/.

## Ethics Statement

The studies involving human participants were reviewed and approved by the Case Western Reserve University Institutional Review Board, DHHS FWA00004428 and IRB registration number 00000683. The participants provided their written informed consent to participate in this study.

## Author Contributions

Both authors created the experimental design and the neutral and moral scenarios used in the materials and wrote the manuscript. JH recorded the audio stimuli and prepared the manuscript for submission. FP coordinated video production/editing, prepared the surveys for data collection, collected the data, and performed the statistical analysis.

## Conflict of Interest

The authors declare that the research was conducted in the absence of any commercial or financial relationships that could be construed as a potential conflict of interest.

## Footnotes

1 Other preference statements used personal pronouns, e.g., *Shelley was saying if we can clone humans, we can fix genetic disorders and end suffering. Alicia was saying there’s never a good reason to go down that path. It’s tough to say, but I guess I agree with her*. Here, the third person pronoun *her* could refer to either of the two underlined referents.

2 While maintaining a fairly constrained template for the preference statement (see full stimuli in Supplementary Material), 2 of the 3 preference statements we used included a second hedge within the preference statement, e.g., HEDGE + *I guess I agree with her*. Given the results of recent corpus studies (Hinnell, 2019, 2020) and current research on “stance stacking,” which has shown that most frequently, highly stanced elements co-occur with each other (i.e., are “stacked,” Dancygier, 2012), we incorporated this type of more natural speech in our stimuli.

3 We refer to the two statements throughout the paper as A and B statements to be consistent between moral and neutral trials (i.e., neutral trials have no morally unacceptable or acceptable positions). In cases where we discuss moral scenarios only, we will use morally unacceptable (A) and morally acceptable (B) for ease of comprehension.

4 As one reviewer pointed out, this could be because gesture was not particularly salient, however, this could also be because participants thought gesture was so central to the study it did not bear mentioning. A more structured debriefing would help us evaluate this for future studies.
