# Corrigendum: Gesture Influences Resolution of Ambiguous Statements of Neutral and Moral Preferences

**DOI:** 10.3389/fpsyg.2021.664194

**Published:** 2021-03-05

**Authors:** Jennifer Hinnell, Fey Parrill

**Affiliations:** ^1^Department of English Language and Literatures, The University of British Columbia, Vancouver, BC, Canada; ^2^Department of Cognitive Science, Case Western Reserve University, Cleveland, OH, United States

**Keywords:** cohesive gesture, co-speech gesture, reference resolution, preference, contrast, discourse, multimodal communication, moral issues

In the original article, there was a mistake in Figure 1 as published. Figure 1 contains images reproduced from a television program that are part of the Red Hen dataset, available at redhenlab.org. As Frontiers does not apply Fair Use, the image has been removed from the figure, and a link has been inserted for readers to view the image on a public website. The corrected Figure 1 appears below.

**Figure 1:**
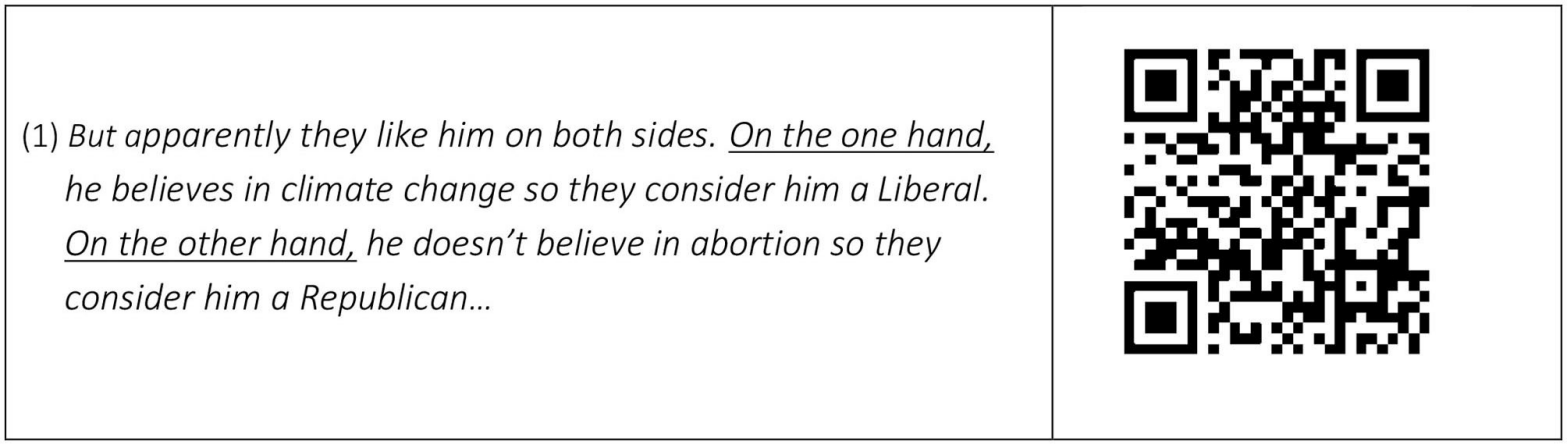
Contrastive use of gesture. 2015-09-24_1700_US_KABC_The_View, 191-201. Red Hen dataset http://redhenlab.org (click here or scan QR code to view the video clip; Uhrig, 2020).

Additionally, in the original article, Uhrig (2020) was not cited. The citation has now been inserted in Figure 1, as shown above, and the corresponding reference has been added to the reference list.

The authors apologize for these errors and state that they do not change the scientific conclusions of the article in any way. The original article has been updated.
