# Supplementary material for: Gesture Influences Resolution of Ambiguous Statements of Neutral and Moral Preferences
Source: Front Psychol. 2020 Dec 10;11:587129. doi: 10.3389/fpsyg.2020.587129 (PMC7758198; doi:10.3389/fpsyg.2020.587129)
Supplement: Supplementary file 1 [file Data_Sheet_1.PDF]

Supplementary Material

Integrating gestures into understanding of neutral and moral preferences

Appendix 1: Demographic details

Item	Proportion
What do you think study was about: Proportion that mentioned gesture/body language/action	.08
Gender, proportion female*	.49
Age (mean)	36.7
Proportion fluent in an additional language (all were native speakers of English)	.52
Political ideology: Proportion that identified as…	
Progressive	.34
Conservative	.58
Chose not to say	.08
Race/Ethnicity: Proportion that identified as….	
American Indian or Alaskan Native	.01
Asian	.03
Black or African American	.05
Native Hawaiian or Other Pacific Islander	0
White	.82
Multiracial	.09
Chose not to say	0

*Choices were male/female/other, no participant selected other

Appendix 2: Stimuli

Table A2.1 shows the scenarios we used in the study. The second column presents two different kinds of data. For neutral scenarios, we show the proportion of participants who chose the A statement as their own preference in our previous work. For moral scenarios, we show the proportion who believed that a position related to the topic used in the scenario was morally unacceptable. The final column shows the proportion of participants who chose the A statement as their personal preference/ opinion in the current study. The A statement was always the morally unacceptable position. Thus, for neutral scenarios, both columns should show a proportion close to .50, and for moral scenarios, both columns should show a proportion lower than 30%.

Table A2.1

Scenarios

Num.	Proportion chose A in previous study/morally unacceptable	A Statement	B Statement	Hedge	Preference Statement	Prop choosing A
1	0.6	Lauren likes those automatic hand dryers because they are more energy efficient	Kira hates them because they are so loud	I get both their points, but	I think she's right.	0.58
2	0.6	Molly thinks people shouldn't say they have food allergies just because they don't want to eat certain things	she also knows people say they are allergic when they aren't technically, because they really don't feel good if they eat dairy or whatever	I don't know, but	I guess I agree more with that.	0.5
3	0.6	José thinks shopping at independent stores is better for the environment	Ahmil thinks big box stores are better because you make less trash from packaging.	it's tough to say, but	I think he's right.	0.69
4	0.52	A lot of candidates memorize portions of their speech instead of speaking naturally	some candidates think speaking freely sounds better.	I can see what they're getting at, but	I think they're right.	0.24
5	0.48	Jason likes to take the train to games so we don't have to pay for parking	Sammy likes to drive because it's a lot faster	hard to know, but	his argument makes more sense to me.	0.31
6	0.44	My little brother's not on facebook because he thinks its a waste of time	My other brother says he can't do job networking without it	I can see what they're getting at, but	I guess I agree with him.	0.42
7	0.44	Lawn care experts say you should leave the clippings on the lawn because it's good for the grass	people say you should bag the clippings because it looks messy.	it's tough to say, but	I guess I agree with them.	0.43

8	0.4	My grandma always puts off calling the doctor as long as she can	My grandpa calls right away just in case	they both have their reasons, but	that makes more sense to me.	0.37
9	0.4	Sarah says you shouldn't call yourself a Christian if you don't ever go to church	Maria says lots of Christians have a more personal relationship to God	hard to know, but	I guess that makes more sense.	0.14
10	0.4	Mark always goes to pick up the pizza because it's way faster	Miles would rather wait and not have to leave the house	they both have their reasons, but	I guess I agree with him.	0.33
11	0.36	Some people think if you give poor people money without conditions they will spend it on drugs or liquor	Many people think they'll spend it on food and rent and education	I don't know, but	I guess I agree more with that.	0.24
12	0.36	A lot of university students are really specific in what kind of person they will date	Others say they're open to dating all kinds of people	I get both their points, but	I guess that makes more sense.	0.46
13	0.09	My best friend Junie swears that having affairs keeps her marriage more interesting	My friend Emily who's a therapist says affairs really pull a marriage apart.	I get both their points, but	I think she's right.	0.19
14	0.09	Some studies say people can have affairs and still have happy marriages	other studies say affairs really undermine trust in a marriage	I don't know, but	I guess I agree more with that.	0.24
15	0.17	Rachel thinks having more than one wife is fine as long as the wives all agree to it	Naomi says polygamy is based in inequality so it's wrong	it's tough to say, but	I think she's right.	0.36
16	0.17	Aisha pointed out that wives in polygamous households get to share all the childcare, which makes life easier	her sister says that doesn't make up for looking after your husband's children that aren't yours.	I can see what they're getting at, but	I think she's right.	0.6
17	0.23	My grandmother said it's fine for kids to be overweight - it's just baby fat and they grow out of it.	my mom says she'd really worry about long term health effects if I weighed too much	I can see what they're getting at, but	that makes more sense to me.	0.19
18	0.26	My rich friend Brian thinks it would be good to be poor because you can't be materialistic if you don't have money	my friend Shane who's a social worker says he sees a lot of negative effects to people's health and life expectancy due to poverty,	I can see what they're getting at, but	I guess I agree with him.	0.25
19	0.27	Kim said some teens can be just as good mothers as women in their twenties and thirties	Tina said if you're not old enough to drive you shouldn't be old enough to be a mom	it's tough to say, but	I guess I agree with her.	0.37
20	0.15	Melanie said slavery was good because it built the US economy and we all benefit from that	her sister said how can something be good that relied on forced labor	they both have their reasons, but	that makes more sense to me.	0.28

21	0.15	Angie said that some slave owners tried to treat their slaves with humanity	Artie pointed out that, regardless, the slaves were still someone's property and that's immoral	hard to know, but	I guess that makes more sense.	0.26
22	0.22	Some activists say vandalism as a form of political protest is sometimes necessary	others say destroying property is never the right thing to do.	they both have their reasons, but	I guess I agree with them.	0.39
23	0.22	Reese argues that spraying graffiti on businesses that do bad things is an important way to have a voice	George made the point that adding crime to bad behavior doesn't fix anything	I don't know, but	I guess I agree more with that.	0.43
24	0.14	My niece says cloning humans is a necessary scientific step	my mom says it's something science should just never do.	I don't know, but	I think she's right.	0.49
25	0.17	Some people say drug addiction isn't a problem since people are really just hurting themselves	other people do think it's a problem because drug addicts are always going to hurt someone else eventually	hard to know, but	I guess I agree more with that.	0.26
26	0.18	My dad thinks suicide should be allowed because it ends suffering	my uncle thinks it should never be allowed because people can always be helped	I can see what they're getting at, but	I think he's right.	0.35
27	0.22	One group of economists says it's good when a lot of people are unemployed because their jobs aren't necessary	another group says our economy needs everyone to be employed	it's tough to say, but	I think they're right.	0.68
28	0.26	My dad says racial prejudice is just part of being human, because we like our own kind best	my mom says racial prejudice can't be excused even if it is human nature	I don't know, but	I guess I agree more with that.	0.26
29	0.27	Todd says air pollution is just part of living in a civilized country	William says civilized societies just find ways to keep the air clean.	I get both their points, but	I guess I agree with him.	0.58
30	0.28	The city council said that having rich and poor neighborhoods in the city is fine, because it's up to people to move if they don't like it	some audience members said that keeping people separated by income is not okay	hard to know, but	that makes more sense to me.	0.39
31	0.14	Shelley was saying if we can clone humans, we can fix genetic disorders and end suffering	Alicia was saying there's never a good reason to go down that path	it's tough to say, but	I guess I agree with her.	0.46
32	0.17	Lorena told me lots of drug addicts are totally functional so we shouldn't worry about them	Seanna made the point that most addicts don't want to be addicts and actually want help	they both have their reasons, but	that makes more sense to me.	0.17
33	0.18	Mike says killing yourself is an understandable response to terrible circumstances	Jon says nobody should ever be allowed to get to that point	hard to know, but	I guess that makes more sense.	0.32

34	0.22	Chris says it's fine if a lot of people are out of work because they can just go on benefits	Malik says he doesn't want to pay taxes so other people don't have to work	they both have their reasons, but	I guess I agree with him.	0.36
35	0.26	A guy at the party said if everyone in a society has racial prejudice, it's not a problem	then the host was like that's only true if everyone in the society has equal power	I get both their points, but	I guess I agree more with that.	0.19
36	0.27	Jonay said if the air in your city isn't clean you should move	Desire says we all need to keep the air clean because what about people who can't just move	I get both their points, but	I guess that makes more sense.	0.08
